# Analysis of recombinant proteins for Q fever diagnostics

**DOI:** 10.1038/s41598-020-77343-0

**Published:** 2020-12-01

**Authors:** Halie K. Miller, Gilbert J. Kersh

**Affiliations:** Rickettsial Zoonoses Branch, Centers for Disease Control and Prevention, Atlanta, GA USA

**Keywords:** Microbiology, Bacteria

## Abstract

Serology is essential for Q fever diagnostics, a disease caused by the bacterial pathogen *Coxiella burnetii.* The gold standard test is an immunofluorescence assay utilizing whole cell antigens, which are both dangerous and laborious to produce. Complexities of the antigen coupled with the subjective nature of the assay lead to decreased uniformity of test results and underscore the need for improved methodologies. Thirty-three *C. burnetii* proteins, previously identified as immunoreactive, were screened for reactivity to naturally infected goat serum. Based on reactivity, 10 proteins were analyzed in a secondary screen against human serum from healthy donors. Assay sensitivity and specificity ranged from 21 to 71% and 90 to 100%, respectively. Three promising antigens were identified based on receiver operating characteristic curve analysis (CBU_1718, CBU_0307, and CBU_1398). Five multiplex assays failed to outperform the individual proteins, with sensitivities and specificities ranging from 29 to 57% and 90 to 100%, respectively. Truncating the top antigen, CBU_1718, had no effect on specificity (90%); yet sensitivity decreased dramatically (71% to 21%). Through this study, we have expanded the subset of *C. burnetii* immunoreactive proteins validated by enzyme-linked immunosorbent assay and demonstrate the effect of novel antigen combinations and protein truncations on assay performance.

## Introduction

Diagnostic testing for Q fever, caused by the bacterial pathogen, *Coxiella burnetii,* is largely dependent on serology^1,2^. During the early stages of infection, a diagnosis can be made through detection of bacterial DNA by PCR from samples of whole blood or serum; however, the window of effectiveness is usually limited to the first two weeks of symptom onset^2^. Symptoms of acute Q fever are often nonspecific with fever, fatigue, chills and myalgia being the most frequently reported^2^. This often leads to a low clinical suspicion, which limits the ability to effectively utilize PCR-based assays. Due to the persistence of IgG antibodies to *C. burnetii* for months to years after infection, serological diagnosis of acute Q fever relies on paired acute and convalescent samples taken 3–6 weeks apart^2^. In less than 5% of acute cases, chronic Q fever can develop with symptoms ranging from endocarditis to chronic hepatitis or chronic vascular infections^2^. The success of PCR in diagnosis of chronic Q fever cases ranges from 33 to 64%; as a result, serology is essential for Q fever diagnostics. Persistent IgG antibodies can also influence serological testing for chronic Q fever; therefore, serology is not reliable in the absence of clinical findings. The performance of serology-based testing methods can vary based on the source of the antigen, background titers of the population being tested, as well as the type of test being utilized^3^.

Methods for serological testing include complement fixation, radioimmunoassay, enzyme-linked immunosorbent assay (ELISA), Western blotting and the immunofluorescence assay (IFA). IFA is the gold standard serologic assay for Q fever, and in the United States, more than 12,800 serum samples are tested annually by this method^2,4^. The assay utilizes antigen coated onto slides to detect the presence of anti-*C. burnetii* IgG antibodies from serum resulting in fluorescence that is visualized under a microscope, which can be subjective. The antigens used are inactivated cells from the Nine Mile Phase I and Nine Mile Phase II isolates of *C. burnetii*. Nine Mile phase I is the *C. burnetii* reference strain and IgG antibodies against this strain typically develop in higher abundance in chronic Q fever patients^2^. The phase II strain of *C. burnetii* is an avirulent form of the Nine Mile strain and IgG antibodies against it typically develop to higher levels during acute Q fever^2^. Production of the phase I antigen is hazardous owing to the need to culture large amounts of pathogenic bacteria, which requires specialized equipment in a BSL3 facility^2,5^. *C. burnetii* has a low infectious dose of 1–10 organisms, is naturally stable in the environment for long periods, is able to spread via aerosols, and has been previously weaponized^6,7^. These characteristics led to its classification as a potential bioweapon and a select agent, which further restricts antigen preparation to select facilities^8^. The slow growth kinetics of the bacteria coupled with its specialized growth requirements are additional barriers to large-scale production. Furthermore, the inherent complexities of the whole cell antigen as well as the subjective nature of the IFA lead to inconsistencies in test results.

A recombinant protein based diagnostic assay would eliminate the need for the dangerous and labor-intensive generation of whole cell antigen and improve test uniformity. A number of studies have identified *C. burnetii* immunogenic proteins using a variety of methodologies including 2-dimensional gel electrophoresis (2D-GE), protein microarrays and even immunocapturing^9–26^. Only ~ 20% of the identified *C. burnetii* immunoreactive proteins have been validated in an ELISA; none of which are as sensitive and specific as the whole cell antigen^13,14,16,20,27^. This suggests a need for further exploration of the utility of these antigens in a multiplexed assay, which has not been developed. This study aims to expand the subset of *C. burnetii* immunoreactive proteins that have been validated by ELISA and assess the top candidates for use in a multiplexed assay. Furthermore, truncated fragments of the best antigen (CBU_1718) were generated in an effort to improve antibody detection.

## Methods

### Bacterial strains, plasmids and growth conditions

The *Coxiella burnetii* strain used in this study was Nine Mile phase I (NMI)^28^. This strain, isolated from a tick in 1935^1^, can cause Q fever in humans^29^, and it is the reference strain used as whole cell antigen in current diagnostic tests. The strain was grown in ACCM-2 and DNA was extracted as previously described^30^. The *Escherichia coli* strain used in this study was K12 JM109 (New England Biolabs). Plasmid pIVEX2.4d was utilized as the over-expression vector (Biotechrabbit). The recombinant *C. burnetii* constructs used in this study are listed in Supplemental Table 1. *E. coli* was grown in Luria–Bertani medium at 37 °C. When required, carbenicillin was used at 100 mg l^−1^.

### Over-expression and purification of recombinant proteins

Coding regions of each of the 33 proteins and 4 truncations were amplified from the *C. burnetii* NMI template by PCR using gene specific primers (Table S1). PCR reactions were carried out using Phusion High-Fidelity DNA Polymerase (Thermo Fisher Scientific) per the manufacturer’s instructions. Resulting PCR products were cloned into the over-expression vector pIVEX2.4d, introducing an N-terminal 6 histidine tag. The recombinant *C. burnetii* constructs were purified using the HiSpeed Plasmid Midi Kit (Qiagen) per the manufacturer’s instructions and confirmed by sequencing. Cell-free expression of the recombinant proteins was performed using the RTS 100 *E. coli* HY kit (Biotechrabbit) according to the manufacturer’s protocol. Proteins for the primary screen were purified using Ni–NTA magnetic agarose beads (Qiagen) under native conditions and stored in 25% glycerol at − 80 °C. Large scale expressions were performed for the secondary screen using the RTS 500 ProteoMaster *E. coli* HY Kit (Biotechrabbit) and purified using Ni–NTA agarose (Qiagen) via the column method under native conditions. Post purification buffer exchange and concentration was performed using Amicon Ultra-15 Centrifugal Filter Units (Millipore Sigma) per manufacturer’s instructions. Purified proteins were stored at − 20 °C in PBS containing 1 × protein stabilization cocktail (Thermo Fisher Scientific). Protein expression was verified by SDS-PAGE analysis.

### Serum sample procurement

Goat serum samples for the primary screen were from stored stocks obtained during a 2011 Q fever outbreak^31^. For the secondary screen, we utilized de-identified and banked human serum samples that were originally derived from routine blood donations in the United States. Serum samples were considered positive for anti-*C. burnetii* antibodies against either phase I or phase II *Coxiella* based on IFA (titer ≥ 16) and/or ELISA (absorbance ≥ twofold relative to BSA controls). Samples were stored at − 80 °C. This study does not involve human subjects under 45 CFR 46.102(f) and was approved by the Human Research Protection Office of the Centers for Disease Control and Prevention.

### Enzyme-linked immunosorbent assay (ELISA)

Proteins were coated onto immulon 2 high bind 96-well microtiter EIA plates (Thermo Fisher Scientific) at 2 µg ml^−1^ by incubating at 4 °C in 100 µl per well of coating buffer (50 mM Na_2_CO_3_, 50 mM NaHCO_3_, pH 9.6) overnight. Empty well controls contained coating buffer only. Plates were washed in 300 µl per well of PBS containing 0.05% Tween-20 (PBST). Wells were blocked with 200 µl for 2 h at 37 °C in a humidified atmosphere. Assays using goat serum were blocked in PBST containing 5% bovine milk; assays using human serum were blocked in 50% PBST/50% SEA block buffer (Thermo Fisher Scientific). Following the blocking step, plates were incubated with 100 µl per well of serum (1:100) in PBST for 1 h at 37 °C in a humidified atmosphere. Plates were washed and incubated with 100 µl per well of a biotin-labeled, species-specific primary antibody against IgG (KPL) at 1:50,000. Plates were washed and incubated with 100 µl per well of peroxidase-labeled streptavidin (KPL) at 1:2000 for 1 h each at 37 °C in a humidified atmosphere. Detection was achieved through addition of 100 µl per well of ABTS substrate (KPL). Reactions were stopped with 100 µl per well of 1% SDS and absorbance measured at 405 nm using an ELx800 microplate reader (BioTek).

### Statistical analysis

Sensitivity and specificity were determined by receiver operating characteristic (ROC) curve analysis. A 5% confidence limit was used to determine statistical significance. All analyses were performed using GraphPad Prism version 7.01 (GraphPad Software Inc., La Jolla, California).

### Disclaimer

The findings and conclusions in this report are those of the authors and do not necessarily represent the views of the CDC.

## Results

### Selection and primary screening of immunoreactive proteins

A meta-analysis was performed to find studies between 2005 and 2015 that identified candidate *C. burnetii* immunoreactive proteins. A total of 16 publications were found with methodologies for seropositive identification including 2-dimensional gel electrophoresis, Western blot and protein microarrays^11–26^. Proteins selected for inclusion in this study fall into three categories. Category I includes 13 proteins based on seroreactivity in ≥ 4 publications. Category II includes three proteins, which demonstrated seroreactivity against human serum in ≥ 3 studies. Finally, category III consists of 17 proteins all of which were identified in ≥ 2 immunoproteomic studies, with at least one of those studies using human serum.

**Figure 1:**
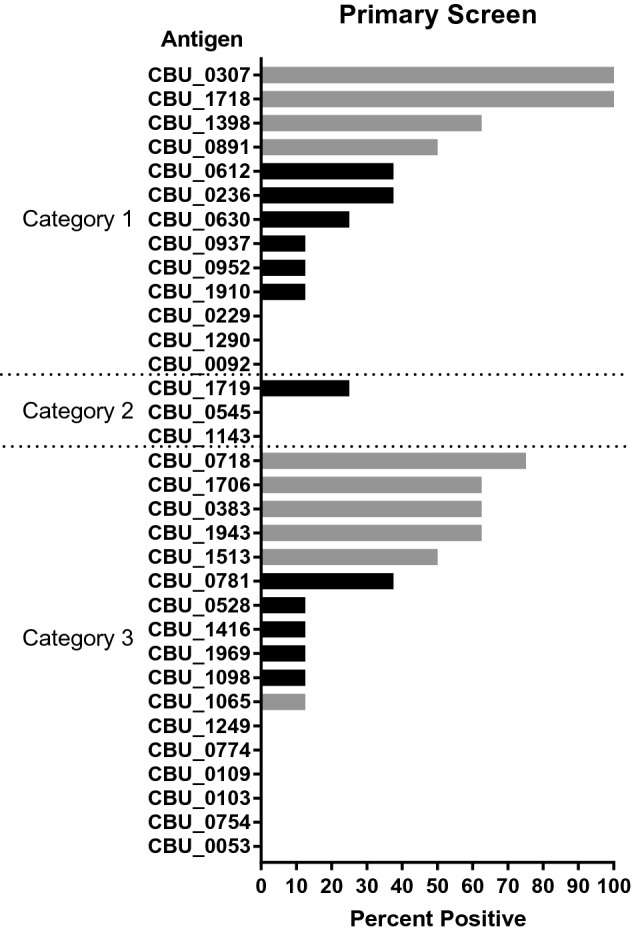
Primary screen of recombinant protein antigens. Recombinant proteins were used as antigens in enzyme-linked immunosorbent assays (ELISA) against 8 goat serum samples that were positive for antibodies against phase I and/or phase II whole cell *C. burnetii*. Positive reactions were defined as an absorbance reading (405 nm) ≥ mean + 3 × the standard deviation of BSA negative controls and demonstrated statistical significance (p-value < 0.05) based on a Student’s t-test. Data is presented as the percentage of positive serum samples for each antigen. Grey bars represent recombinant protein antigens that were selected for further assay development.

A total of 33 proteins were used as antigens in ELISAs to assess reactivity to goat serum positive for antibodies against phase I and/or phase II *C. burnetii*. Goat serum was utilized in the initial screen in order to preserve limited stocks of anti-*C. burnetii* antibody positive human serum. Positive reactions were defined as an absorbance reading (405 nm) ≥ mean + 3 × the standard deviation of BSA negative controls and demonstrated statistical significance (p-value < 0.05) based on a Student’s t-test. A total of nine proteins were selected for further analysis based on the ability to detect antibodies against *C. burnetii* in ≥ 50% of the serum samples tested (Fig. 1). Although, CBU_1065 was only able to detect antibodies in 12.5% of the serum samples tested, the intensity of the reaction was much greater than other antigens. Therefore, it was also included in additional screening as it might be useful in improving sensitivity of a multiplexed assay. The remaining proteins were considered weakly reactive (< 50% positive) or nonreactive (0% positive) and were excluded from further analysis.

### Secondary screening of immunoreactive proteins

**Figure 2:**
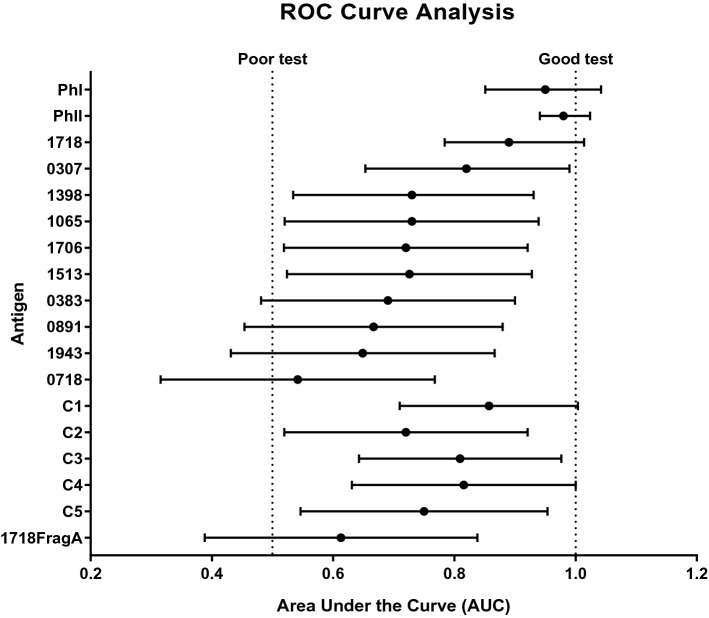
ROC curve analysis of recombinant protein antigens. Recombinant proteins were used as antigens in enzyme-linked immunosorbent assays (ELISA) against 24 human serum samples. Fourteen were positive for antibodies against phase I and/or phase II; 10 were negative. The AUC for each antigen is displayed as black dots, bars represent the 95% confidence interval.

The top 10 immunoreactive proteins selected from the primary screen were utilized as antigens in ELISAs to assess their ability to correctly differentiate human serum positive for anti-*C. burnetii* antibodies against negative controls. The recombinant proteins were compared to whole cell phase I and phase II *C. burnetii* using a panel of 24 human serum samples (14 positives by IFA for either phase I or phase II anti-*C. burnetii* antibodies; 10 samples negative by IFA). ROC curve analysis was performed using the resulting absorbance readings to determine the accuracy of each protein as a diagnostic antigen (Fig. 2). The area under the ROC curve (AUC) determines how well the diagnostic test can classify serum as containing anti-*C. burnetii* antibodies. An AUC of 1 indicates an accurate test; whereas, a test with an AUC of ≤ 0.5 is unable to distinguish the positive samples from the negative controls. The best antigens, CBU_1718 and CBU_0307, were able to correctly classify the same number of serum samples (71.43% sensitivity and 90% specificity) (Table 1). CBU_1718 had a slightly better discriminative ability (AUC 0.8786, p = 0.0019) compared to CBU_0307 (AUC 0.7929, p = 0.0164). Additionally, CBU_1398 had an AUC of 0.75 and showed significant discriminative ability (p < 0.05); however, the sensitivity was only 57%. No other recombinant protein antigens tested had significant discriminative ability. The whole cell antigens, which are the gold standard for diagnostic testing and were the antigens used in the IFA to define the true positive samples in this study, outperformed all of the tested proteins with a sensitivity of 85.71% and 100% specificity for PhI (AUC 0.95, p = 0.0002) and 92.86% sensitivity, 100% specificity for PhII (AUC 0.9786, p < 0.0001). The most inaccurate protein was CBU_0718 with a sensitivity of 28.57% and a specificity of 90% (AUC 0.5786, p = 0.5195).

**Table 1:** Specificity and sensitivity of recombinant proteins.

Antigen	Specificity (%)	Sensitivity (%)	Antigen combinations				
			C1	C2	C3	C4	C5
PhI	100	85.71					
PhII	100	92.86					
1718	90	71.43	X	X	X	X	X
0307	90	71.43	X			X	X
1398	90	57.14	X	X	X		
1065	90	50	X	X			
1706	90	50					
1513	90	35.71					
0383	90	35.71					
0891	90	28.57	X	X			
1943	90	21.43					X
0718	90	28.57					
C1	100	57.14					
C2	90	50					
C3	100	50					
C4	90	42.86					
C5	90	28.57					
1718FragA	90	21.43					

### Analysis of multiplexed antigens on assay accuracy

Multiplexed assays were performed using combinations of protein antigens to improve assay sensitivity. We tested five combinations based on predicted sensitivity and specificity from the singleplex ROC curve analysis. As demonstrated in Table 1, combination 1 equally combined five different proteins (CBU_1718, CBU_0307, CBU_1398, CBU_1065 and CBU_0891) and performed the best with a sensitivity of 57.14% and specificity of 100%. The AUC of combination 1 was 0.85, p = 0.0041, while still significant, it was worse than the top performing single antigen, CBU_1718 (Fig. 2). Combinations 3 (AUC 0.8286, p = 0.0071) and 4 (AUC 0.7786, p = 0.0224) both had significant discriminative ability with sensitivities/specificities of 50%/100% and 42.86%/90%, respectively. Combinations 2 (AUC 0.7286, p = 0.0610) and 5 (AUC 0.7, p = 0.1011) demonstrated sensitivity/specificity of 50%/90% and 28.57%/90%, respectively. For each of the five combinations none was more accurate than the individual proteins.

### Analysis of protein truncations on assay accuracy

We examined the efficacy of truncated proteins as antigens in an effort to eliminate potential non-specific epitopes and improve assay specificity. We selected the best antigen based on the results of the secondary screen, CBU_1718, for this analysis and created four fragments by eliminating portions of either amino-or carboxy-termini at random (Table 2). Fragment A is 27 kDa and lacks the amino-terminal. The carboxy-terminal was eliminated from truncations B, C and D, which are 32 kDa, 49 kDa and 53 kDa, respectively. Protein fragments were analyzed against six serum samples selected based on results from the full-length CBU_1718 protein antigen in the secondary ELISA screen (Table 2). Three serum samples were used that were negative on the IFA against the PhI and/or PhII whole cell antigen. The true negative was also negative using the full-length CBU_1718 antigen, borderline negative was close to the cut-off value in an ELISA with the full-length CBU_1718, and false positive was positive using the full-length CBU_1718 antigen due to non-specific binding. Three serum samples were tested that were positive against the PhI and/or PhII whole cell antigen by IFA. The false negative was negative by ELISA using the full-length CBU_1718 antigen, borderline positive was close to the cut-off, and the last was a strong positive based on results of the full-length CBU_1718 protein in the secondary ELISA screen. Fragments B and C lost reactivity to the borderline positive and strong positive serum samples; however, the false positive serum sample was still recognized by these fragments. The third C-terminal truncation, fragment D did not lose reactivity to any of the tested serum samples. Interestingly, fragment A lost reactivity to the false and borderline positives; yet, the strong positive was still recognized. Therefore, we analyzed fragment A against all 24 serum samples in order to determine how it performs as an antigen relative to the full-length protein (Table 1 and Fig. 2). Although specificity of the fragmented antigen did not change (90%), assay sensitivity fell to 21.43% with an AUC of 0.5714 (p = 0.5582).

**Table 2:**
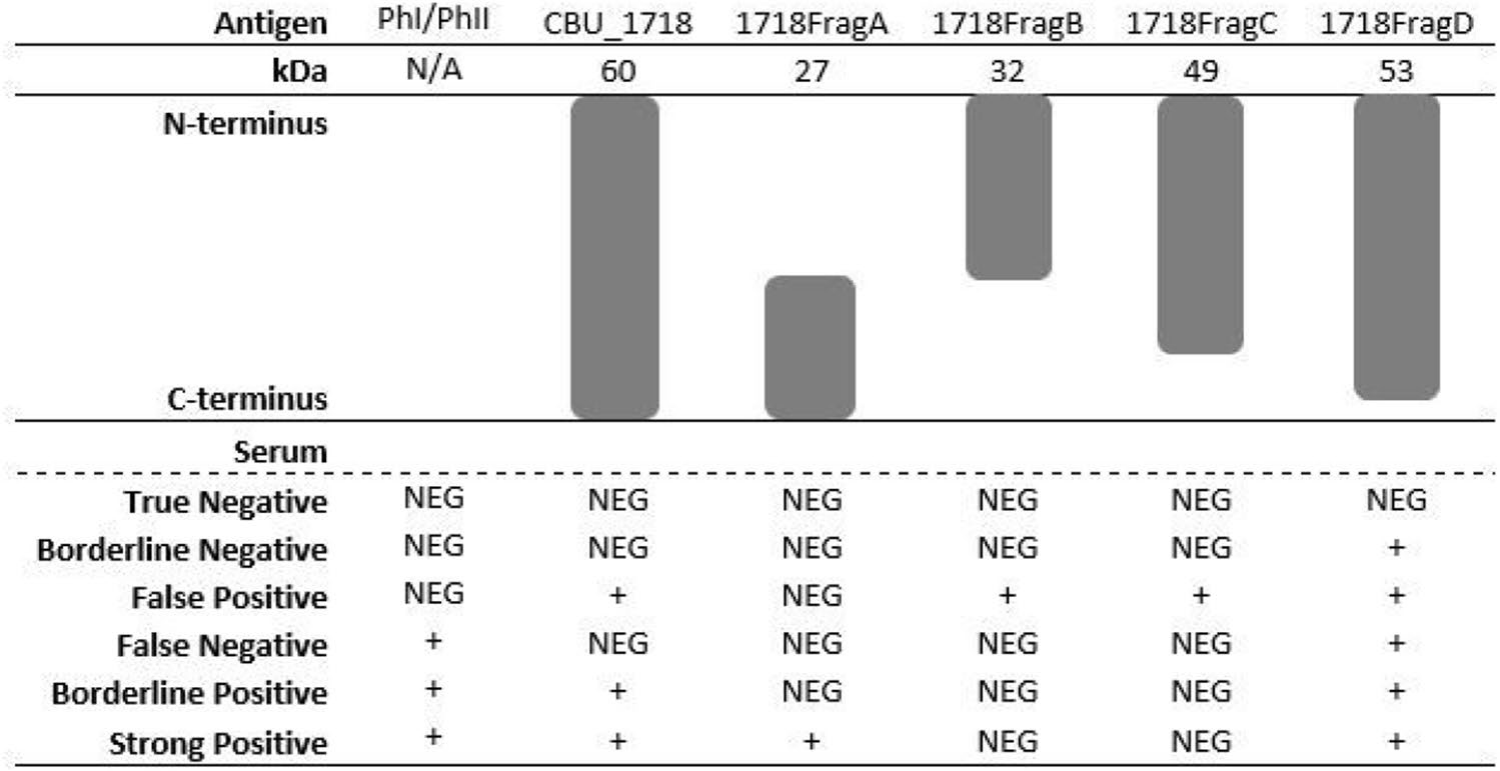
Analysis of protein truncations.

## Discussion

A total of 33 *C. burnetii* recombinant proteins were analyzed for use as antigens in singleplex and multiplex ELISAs in an effort to improve Q fever diagnostic testing. All tested proteins had previously been identified as immunoreactive, 17 of which had never been validated by ELISA^11–26^. In a primary screen against sera from naturally infected goats, 9 proteins displayed reactivity to greater than 50% of the samples tested and were selected for further assay development. In a secondary screen against human sera from healthy donors, assay sensitivity ranged from 21 to 71% for the individual proteins with specificities ranging from 90 to 100%. Three of the tested recombinant proteins were good candidates based on ROC curve analysis.

Eleven of the tested proteins had no reactivity in the initial screen while 13 were considered weakly reactive. One protein found to be weakly reactive in the present study was the highly published antigen, CBU_1910 (Com1)^32^. Com1 had previously been identified as seroreactive against human and rodent sera with sensitivity and specificity ranging from 37.5% to 100% and 71% to 100%, respectively^13,33,34^. Interestingly, this antigen performed poorly in this study when tested against serum from naturally infected goats. These findings suggest that the antigenicity of Com1 and possibly other antigens may be species-specific. Similarly, the three proteins selected for inclusion based on category II criteria (≥ 3 human serum studies) were weakly reactive (CBU_1719) or non-reactive (CBU_1143 and CBU_0545) against goat serum in the initial screen. The latter proteins have only been identified in screens using human serum; whereas CBU_1719 was identified in screens using either human or rodent serum. In a comprehensive review of *C. burnetii* immunoreactive proteins, it was demonstrated that 65% of antigens identified with human serum do not overlap with antigens identified in screens using rodent serum^32^. The findings highlight an important limitation to the study herein, which may have missed useful human diagnostic antigens as a result of using caprine serum for the initial screen. Unfortunately, the use of goat serum was necessary to conserve the limited stocks of human serum while screening a large number of recombinant antigens for functionality.

Combinations of the top performing antigens were attempted by equally pooling from two to five proteins. In each case, the top performing antigen of the mix functioned better independently (Table 1 and Fig. 2). A previous report noted that a *C. burnetii* recombinant protein-based multiplexed assay had been attempted; however, details of the assay, including the antigens used, were not published. In the study, the use of multiple proteins increased assay sensitivity albeit at the expense of assay specificity^13^. In the study herein, assay sensitivity was compromised by combining multiple antigens. This is likely a result of the different analysis performed to determine cut-off values. Herein ROC curves were utilized to assign cut-off values, which allows for the highest true-positive rate while maintaining the lowest false positive rate. In each case, assay sensitivity could be improved by adjusting the cut-off; however, as assay sensitivity increases it does so at the expense of assay specificity. Regardless of the type of analysis used, in the absence of improved antigen specificity, it is unlikely that a multiplexed antigen will surpass whole cell bacteria as the favored diagnostic antigen.

The protein CBU_1718 (GroEL or HtpB), is a heat shock protein and was the best performing antigen among the 33 tested. It was previously identified as an immunodominant *C. burnetii* protein in more than 16 publications against human, goat, mouse and guinea pig sera^27,32^. As one of the most highly published antigens, it is not surprising that a sensitivity of 71% and 90% specificity was identified for the antigen. The usefulness of GroEL as a diagnostic antigen for detecting *C. burnetii* infection in goats was demonstrated previously against both primary and recurrent infections^27^. GroEL has been identified as both immunoreactive and a useful diagnostic antigen in many other organisms including *Burkholderia mallei*, *Burkholderia pseudomallei* and *Helicobacter pylori* among others^35–37^. A previous study utilizing protein microarray demonstrated that GroEL of *C. burnetii* displayed some cross-reactivity as it recognized 20% of sera from persons with rickettsial spotted fever and 10% from persons with *Legionella* pneumonia^23^. Similarly, recombinant GroEL displayed reactivity against 10% of the Q fever negative human serum in the present study. In an effort to reduce this non-specific reactivity, we created truncations of the GroEL protein which has proved useful in other organisms such as PstS1 of *Mycobacterium tuberculosis* or the multi-fragment antigen of infectious bronchitis virus^38,39^. Evaluation of truncated GroEL proteins herein identified a putative *C. burnetii* specific-epitope located between the amino acids 467 to 496 in the C-terminal region. Although sensitivity was vastly decreased herein, the ability to utilize truncated proteins as part of a multiplexed antigen may be a useful tool for further assay development.

Through this study, the subset of *C. burnetii* immunoreactive proteins that have been validated by ELISA has been expanded. Furthermore, the effect of novel antigen combinations and protein truncations on assay performance has been examined. Limitations of the current study include small numbers of serum samples used to screen the antigens. Furthermore, the use of human serum derived from healthy donors limits the ability to determine how these antigens perform against serum from persons with active acute and chronic Q fever.

## Supplementary information

Supplementary Information.
